# Conserved protein Seb1 that interacts with RNA polymerase II and RNA is an antipausing transcription elongation factor

**DOI:** 10.1261/rna.080765.125

**Published:** 2026-01

**Authors:** Krzysztof Kuś, Soren Nielsen, Nikolay Zenkin, Lidia Vasiljeva

**Affiliations:** 1 Department of Biochemistry, University of Oxford, Oxford OX1 3QU, United Kingdom; 2 Centre for Bacterial Cell Biology, Biosciences Institute, Newcastle University, Newcastle upon Tyne NE2 4AX, United Kingdom

**Keywords:** Pol II, transcription termination, CID-RRM factors, elongation factor, Seb1, Scaf4 and Scaf8

## Abstract

Maturation of protein-coding precursor messenger RNA (pre-mRNA) is closely linked to RNA polymerase II (Pol II) transcription. However, the mechanistic understanding of how pre-mRNA processing is coordinated with transcription remains incomplete. Conserved proteins interacting with the C-terminal domain of the largest catalytic subunit of Pol II and nascent RNA (CID-RRM factors) were demonstrated to play a role in pre-mRNA 3′-end processing and termination of Pol II transcription. Here, we use a fully reconstituted system to demonstrate that the fission yeast CID-RRM factor Seb1 acts as a bona fide elongation factor. Our analyses show that Seb1 exhibits context-dependent regulation of Pol II pausing, capable of either promoting or inhibiting pause site entry. We propose that CID–RRM factors coordinate Pol II transcription and pre-mRNA 3′-end processing by modulating the rate of Pol II transcription.

## INTRODUCTION

**FIGURE 1.**
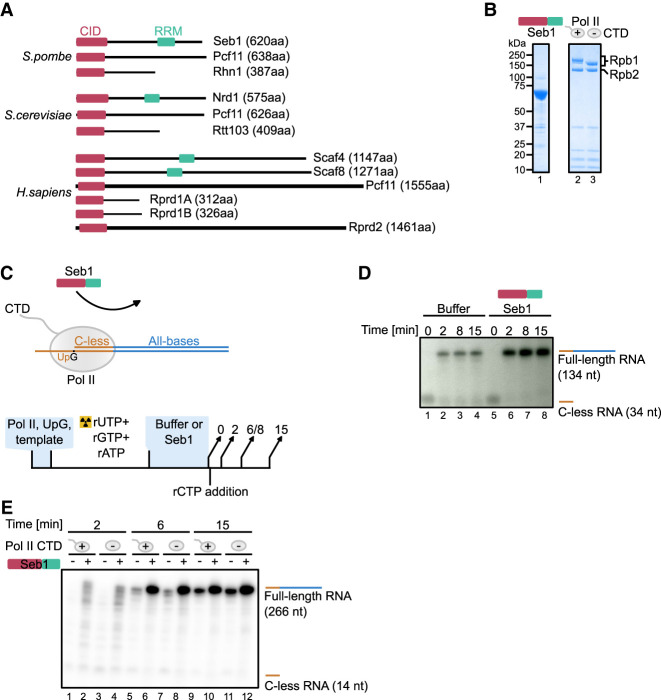
Seb1 stimulates transcriptional output in vitro. (*A*) Seb1 is a conserved protein in eukaryotes. In humans, Seb1 has undergone gene duplication (Scaf4 and Scaf8). This family of proteins contains the CTD interacting domain (CID) and RNA recognition motif (RRM). (*B*) SDS-PAGE showing exemplary purification of Seb1 or Pol II (±CTD). (*C*) Schematics of in vitro transcription, which allows multiple rounds of reinitiation. Tailed DNA template with a single-stranded overhang on the 3′-end of the template strand has a C-less track allowing the elongation complex to stop in the absence of rCTP (*upper* panel). Transcription is primed by an UpG dinucleotide and initiated with α-[^32^P]-UTP, rGTP, rATP. The stopped elongation complex is incubated with or without Seb1 (1.5 µM), and transcription is resumed upon rCTP addition. Products are collected at different time points (*lower* panel). (*D*) Seb1 stimulates transcription in vitro. Marked C-less RNA is 34 nt, whereas extended RNA is 134 nt long. (*E*) Seb1 stimulatory effects on Pol II do not require the CTD. Rpb1 has a TEV-cleavage site, which allows efficient removal of the CTD (Fig. 1B). Marked C-less RNA is 14 nt long, whereas full-length RNA is 266 nt long.

RNA polymerase II (Pol II) is a multisubunit machinery that transcribes all protein-coding and many noncoding RNAs. During Pol II transcription, precursor messenger RNA (pre-mRNA) undergoes three essential processing steps: 5′-end capping, splicing, and 3′-end cleavage/polyadenylation to mature into its functional form. Interactions between Pol II and mRNA processing factors are largely mediated by the C-terminal domain (CTD) of the largest subunit of Pol II (Rpb1). The Pol II CTD is composed of the consensus repeats Y_1_S_2_P_3_T_4_S_5_P_6_S_7_ (29 in fission yeast *Schizosaccharomyces pombe* [*S. pombe*] and 52 in humans) where all residues except prolines can be phosphorylated (Komarnitsky et al. 2000; Buratowski 2003; Eick and Geyer 2013). RNA processing factors show a preference for specific CTD phosphorylation patterns, which is a basis for their stage-specific recruitment or exchange (Buratowski 2003; Hsin and Manley 2012). Among readers of the CTD phosphorylation status are proteins with the CTD-interacting domain (CID). In addition to the CID, some of these proteins also contain an RNA-recognition motif domain (RRM) and are named CID–RRM factors (Fig. 1A). This protein family—which includes *S. pombe* Seb1, Nrd1 in *Saccharomyces cerevisiae* (*S. cerevisiae*) and Scaf4/8 in humans—regulates key transcriptional events, coordinating the 3′-end processing of nascent transcripts and the transition between elongation and termination (Kim et al. 2006; Vasiljeva and Buratowski 2006; Vasiljeva et al. 2008; Grzechnik et al. 2015; Lemay et al. 2016; Wittmann et al. 2017; Gregersen et al. 2019; Kamieniarz-Gdula et al. 2019).

Transcription termination enables the release of RNA and dislodgement of Pol II from the DNA template (Proudfoot 2011, 2016). This process is linked to the 3′-end processing of nascent RNA—where recognition of polyadenylation signal (PAS) motif by the cleavage and polyadenylation factor (CPF) leads to endonucleolytic cleavage of the transcript (for reviews, see Zhao et al. 1999; Proudfoot 2011; Rodríguez-Molina et al. 2023). This cleavage event leaves a downstream, monophosphorylated part of RNA still bound to transcribing Pol II. It provides a window of opportunity for Xrn2/Rat1/Dhp1 to engage and start degrading nascent RNA, which results in Pol II termination (torpedo model) (Kim et al. 2004; West et al. 2004; Eaton et al. 2020). As an alternative, but not mutually exclusive, allosteric model proposes that transcription around the PAS region exerts conformational changes in the Pol II complex aiding termination (Luo and Bentley 2004; Zhang et al. 2015).

The importance of CID–RRM factors is highlighted by their evolutionary conservation with Seb1 homologs present in *S. cerevisiae* and humans (Fig. 1A). Human Scaf4 and Scaf8 are paralogs that have emerged by gene duplication and share ∼40% amino acid identity between them. CID–RRM proteins are essential for viability (Vasiljeva et al. 2008; Lemay et al. 2016; Wittmann et al. 2017; Gregersen et al. 2019). Scaf4 is associated with developmental disorders in humans where one of the alleles is truncated/nonfunctional. This indicates a haploinsufficiency for Scaf4, and expression of both copies is required for proper transcriptional output (Fliedner et al. 2020). Nascent transcriptome analyses upon Scaf8 and Scaf4 depletion indicated that Scaf8 is required for Pol II elongation, whereas Scaf4 is involved in selection of correct PAS by CPF within 3′UTR of the nascent RNA. Seb1 depletion alters transcriptional output, promotes the use of distal polyadenylation sites within 3′UTRs, and impairs transcription termination (Lemay et al. 2016; Wittmann et al. 2017). Moreover, Seb1 was linked to the induction of long-lived Pol II pauses during elongation, which were proposed to contribute to a heterochromatin formation at pericentromeric regions (Marina et al. 2013; Parsa et al. 2018). These observations were based on the analysis of *seb1-1* mutant that has seven-point mutations (three changing amino acids and four silent mutations that affect translation efficiency without changing the amino acid sequence) (Marina et al. 2013).

As mentioned, CID–RRM factors bind to the phosphorylated Pol II CTD, and Seb1 shows a preference for Ser2P but also can interact with Ser5P, whereas Scaf4/8 bind with the highest affinity to dual phosphorylated Ser2P, Ser5P (Becker et al. 2008; Gregersen et al. 2019). This CTD binding bias specifies Seb1 recruitment to the 3′-end of genes where Ser2P CTD phosphorylation peaks (Gu et al. 2013; Kecman et al. 2018; Lyons et al. 2020). Seb1 not only binds to the Pol II CTD but also is in contact with Pol II core (close to RNA exit channel and Rpb4/7) via a region spanning RRM (Kecman et al. 2018). These results were obtained without the presence of nucleic acids indicating a direct interaction between Seb1 and Pol II (Kecman et al. 2018). In addition, it was previously reported that Seb1 is Rpb7 (part of Pol II stalk) binding protein (Mitsuzawa et al. 2003). The putative interface of Seb1–Pol II interaction overlaps with Spt5 (which forms DSIF complex with Spt4) binding site (Bernecky et al. 2017; Ehara et al. 2017; Shetty et al. 2017; Song and Chen 2022). In fact, in vitro data suggested that Seb1 could outcompete Spt4/5 (Kecman et al. 2018). Moreover, Seb1 can interact with RNA close to the promoter region (Lemay et al. 2016; Wittmann et al. 2017) indicating additional roles beyond termination. Further, both activities of Seb1 (CTD binding and RNA recognition) are required for functional transcription. Mutation compromising the CID domain or RRM results in a global transcriptional readthrough in fission yeast (Wittmann et al. 2017). Seb1 associates with the CPF complex implying that CID–RRM factors may directly contribute to pre-mRNA 3′-end processing by CPF leading to changes in PAS usage (Lemay et al. 2016; Wittmann et al. 2017; Gregersen et al. 2019). Notably, Pol II elongation speed has also been linked to PAS selection. Slow Pol II progression favors selection of the more upstream (proximal) PAS, whereas Seb1 depletion favors selection of distal PAS (Pinto et al. 2011; Wittmann et al. 2017; Geisberg et al. 2020; Yague-Sanz et al. 2020).

To unambiguously elucidate the role of CID–RRM proteins in Pol II transcription, we used in vitro biochemistry and bioinformatics to investigate how Seb1 affects Pol II elongation. Our results demonstrate that this conserved factor stimulates Pol II transcription in a defined in vitro system. Comprehensive bioinformatic analysis of the published data sets shows that loss of functional Seb1 alters Pol II pausing genome-wide in agreement with Seb1's role as pro- or antipausing factor. Therefore, we conclude that CID–RRM factors can directly modulate Pol II elongation in addition to playing a role in pre-mRNA 3′-end processing. Our results provide a better understanding of how coupling between transcription and RNA processing is controlled by the CID–RRM factors.

## RESULTS

### Seb1 is an elongation factor in vitro

As Seb1 and its homologs are key to Pol II transcription, we used a reductionist in vitro system to uncover its direct contribution to transcription. To this end, we expressed Seb1 in *Escherichia coli* (*E. coli*) as an N-terminal His-tag fusion. Seb1 was purified using affinity chromatography, followed by gel filtration (Fig. 1B, lane 1). Pol II was obtained from a native source (*S. pombe*) using a strain with a 3xFLAG-tag on Rpb9. Polymerase was purified using affinity (FLAG-M2 agarose resin), followed by ion exchange chromatography (Q column) (Fig. 1B, lane 2). To test how Seb1 contributes to transcription, we used two complementary experimental in vitro strategies. In the first approach, we used a “tailed” DNA template with a single-stranded overhang on the 3′-end of the template strand, thus allowing factor-less transcription initiation with UpG primer. The transcribed sequence started with C-less cassette allowing formation of a halted elongation complex in the absence of rCTP and, thus, synchronization of transcription (Fig. 1C,D, lanes 1 and 5; Supplemental Table 1). The halted elongation complex can then be incubated with factors of interest (Seb1), and transcription resumed by rCTP addition. This setup allows multiple rounds of transcription (Fig. 1C). Addition of Seb1 to the stalled elongation complex had a strong stimulatory effect on further transcription (Fig. 1D, compare lanes 2 and 6). This is surprising considering a previously published observation suggesting that Seb1 is needed to promote Pol II pausing (Parsa et al. 2018).

Given that Seb1 interacts with both the CTD and the core enzyme of Pol II, we investigated whether the CTD is essential for this stimulatory effect. To achieve this, we used a strain with a TEV protease cleavage site upstream of the CTD allowing proteolytic removal of this domain. Pol II–TEV–CTD was purified, treated with either TEV protease or buffer, and further subjected to gel filtration (Fig. 1B, lane 3). Next, the experiment was performed using Pol II with or without this repetitive region (Fig. 1B,E). Both polymerases (±CTD) showed comparable activity (Fig. 1E, compare lanes 5 and 7). Again, we noted that the presence of Seb1 led to increased amounts of full-length RNA (Fig. 1E, compare lanes 5 and 6). Although Seb1 has a CID domain, we have not observed differences in the stimulation of Pol II with or without the CTD (Fig. 1E, compare lanes 6 and 8). Therefore, we conclude that Seb1 does not require the CTD to enhance transcription in this setup.

**FIGURE 2.**
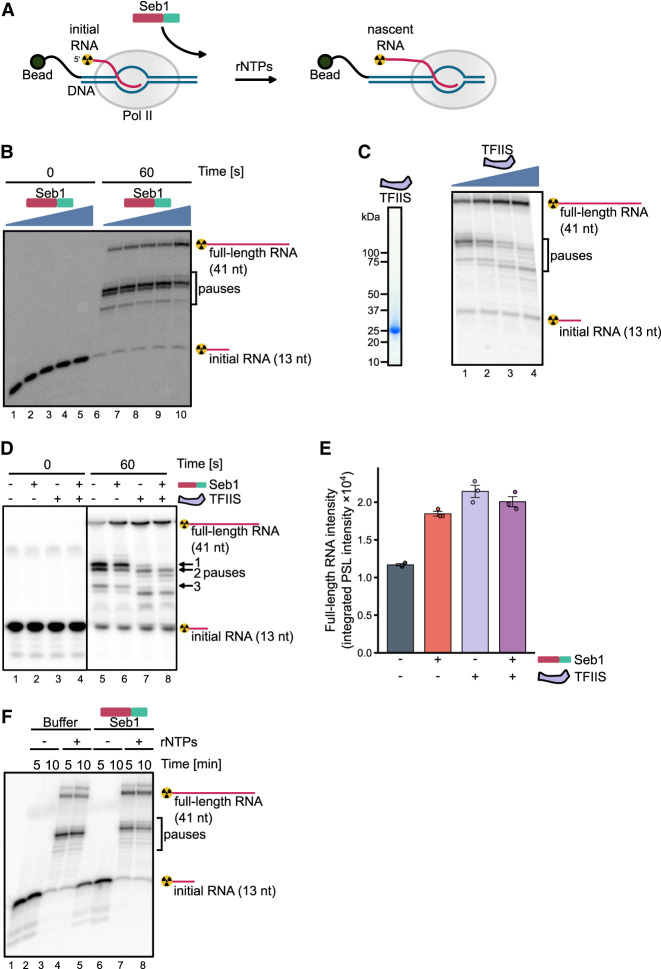
Seb1 affects pausing in vitro. (*A*) Schematic of the in vitro assay with Pol II transcriptional elongation complexes immobilized on streptavidin beads via biotin on the DNA template strand. RNA is ^32^P-labeled at the 5′-end. (*B*) Seb1 decreases pausing in a concentration-dependent manner. The initial RNA has a length of 13 nt and the full-length RNA is 41 nt. Transcription complexes were incubated with 0, 0.5, 1, 2, or 4 µM Seb1, and transcription was initiated with 100 µM rNTPs and 10 mM Mg^2+^. (*C*) The *left* panel shows an SDS gel of purified TFIIS (*S. pombe* TFIIS comprising 115–293 residues). The pausing pattern changes when TFIIS (0, 25, 75, or 240 nM) is present during 30 sec transcription initiated with 900 µM rNTPs and 10 mM Mg^2+^ (*right* panel), indicating backtracking of the elongation complex at these positions. (*D*) TFIIS and Seb1 promote transcription but do not show an additive effect. Transcription complexes were incubated with 1.3 µM Seb1, 60 nM TFIIS, or both, and transcription was initiated with 100 µM rNTPs and 10 mM Mg^2+^. (*E*) Quantification of full-length RNA produced in the experiments in *D*. PSL intensity—photostimulated luminescence intensity. (*F*) Seb1 aids in overcoming TFIIS-sensitive pauses. Transcription complexes were incubated with or without 800 nM Seb1, and transcription was initiated with 900 µM rNTPs and 10 mM Mg^2+^.

As we could not exclude the possibility that Seb1 can act in the dislodgement of Pol II from the DNA template and facilitate another round of transcription, we utilized an alternative in vitro system, using assembled elongation complexes, immobilized on streptavidin beads via biotin on the nontemplate DNA strand (Fig. 2A). Assembled elongation complexes are indistinguishable from native elongation complexes and prevent reinitiation of transcription (Fig. 2B; Supplemental Fig. 1A). Analyses of the in vitro transcription reaction revealed full-length RNA products as well as shorter RNA species corresponding to RNA associated with the paused Pol II. These transcripts represent paused Pol II elongation complexes as they remain bound to the beads via nontemplate strand as can be seen by separating beads and supernatant fractions (Supplemental Fig. 1B). Consistently, Seb1 stimulated RNA extension by Pol II in a concentration-dependent manner and resulted in fewer pauses (Fig. 2B, compare lanes 6 and 10). Pausing can lead to backtracking, when Pol II shifts backward with RNA 3′-terminus leaving the catalytic center via secondary channel. To explore whether some of the pauses that are controlled by Seb1 represent backtracked Pol II, we tested their sensitivity to TFIIS, which stimulates an intrinsic Pol II activity to cleave RNA in the backtracked elongation complex thus reinstating the 3′-end in the active center and reactivating the elongation complex for further RNA extension (Izban and Luse 1992; Reines 1992; Sheridan et al. 2019; Noe Gonzalez et al. 2021). A truncated form of TFIIS lacking domain I, which is nonessential for its activity (Kettenberger et al. 2003), was expressed in *E. coli* and purified to homogeneity, as the full-length protein undergoes degradation (Fig. 2C, left panel). We then evaluated the impact of increasing amounts of TFIIS on the pausing pattern and observed accumulation of full-length RNA accompanied by the simultaneous disappearance of the most prominent pauses, indicating that the observed paused elongation complexes are mainly backtracked ones (Fig. 2C, right panel). This observation led us to investigate whether Seb1 might resolve TFIIS-sensitive pauses. To test this possibility, we performed a side-by-side comparison of Seb1 and TFIIS effects on transcription (Fig. 2D). We observed that both Seb1 and TFIIS result in higher production of full-length RNA (Fig. 2D,E). Interestingly, all three pausing sites that are sensitive to TFIIS are also resolved by Seb1, however with different efficiency (Fig. 2D, lanes 5, 6, and 7). Presence of both Seb1 and TFIIS leads almost to the complete disappearance of pause 1 (Fig. 2D, lanes 7 and 8). We did not observe an additive effect on the accumulation of the full-length RNA product (Fig. 2D, compare lanes 6, 7, and 8 and Fig. 2E), suggesting that pauses are targeted by both factors (though via different mechanisms). However, TFIIS-induced RNA cleavage leads to the formation of new RNA species that are no longer sensitive to either of the factors.

In addition, we performed longer transcription reactions using higher nucleotide concentrations and collected samples after 5 or 10 min. Pausing was decreased only in the presence of Seb1, resulting in an increased amount of full-length product (Fig. 2F, compare lanes 3, 4 with lanes 5, 6). To determine if Seb1 acts to resolve preexisting Pol II pauses or prevents pausing, we performed experiments with the sequential addition of NTPs and Seb1 (Supplemental Fig. 1C,D). Interestingly, there is a stimulatory effect observed when Seb1 is added from the start of transcription elongation suggesting that Seb1 decreases pausing events (Supplemental Fig. 1D). However, although the effect was reduced, Seb1 added at 5 min after the start of transcription elongation also resolved preexisting pauses (Supplemental Fig. 1D). Overall, these results suggest that Seb1 may inhibit Pol II backtracking and/or help resolve nondeeply backtracked Pol II pauses, possibly by interacting with the transcript behind the elongation complex and blocking its reverse movement. Collectively, Seb1 exhibits properties of an elongation factor that can control pausing in vitro.

### Seb1 can lead to different Pol II pausing outcomes in vivo

**FIGURE 3.**
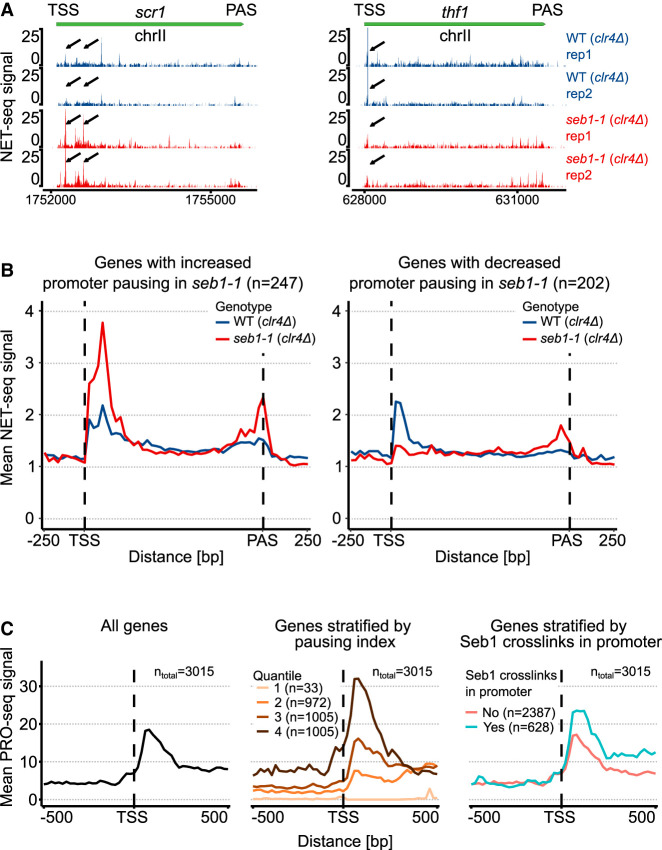
Seb1 correlates with pausing in vivo. (*A*) Seb1 might act as a positive or negative regulator of the Pol II pausing. Two example genes showing the opposite effect on pausing in *seb1-1* cells. (*B*) Metaprofile for the subset of genes exhibiting increased or decreased pausing index in the promoter region (NET-seq) for reference and the *seb1-1* strain. (*C*) Association between Pol II pausing (PRO-seq) and genes with Seb1 cross-linking in the promoter region. The *left* panel depicts PRO-seq signal of all genes, and the *middle* panel presents genes stratified according to the pausing index (quantiles). The *right* panel shows genes grouped based on the presence or absence of Seb1 cross-linking in the promoter region. Total number of genes in each panel *n* = 3015.

Seb1 has been associated with controlling Pol II pausing in vivo (Parsa et al. 2018). It was demonstrated that the *seb1-1* mutant exhibits defects in heterochromatic silencing at the pericentromeric region (Marina et al. 2013). These effects were linked to compromised recruitment of NuRD-related chromatin-modifying complex SHREC and lack of long-lived Pol II pauses in the *seb1-1* mutant as evaluated by native elongating transcript sequencing (NET-seq). It was postulated that Seb1 controls Pol II pausing, which serves as a signal for heterochromatin nucleation (Marina et al. 2013; Parsa et al. 2018). In line with these findings, we decided to revisit NET-seq data to explore different classes of pausing in the *seb1-1* allele (Parsa et al. 2018). Interestingly, the additional analyses of NET-seq data revealed that mutations in Seb1 can lead to either positive or negative effects on Pol II pausing in the promoter region (Fig. 3A). This analysis was performed genome-wide, focusing on protein-coding genes not containing other genes 250 bp upstream of TSS or 250 bp downstream from PAS. We calculated the promoter pausing index for the genes with NET-seq signal (refer to Materials and Methods). Using this approach, we found ∼250 genes that had increased pausing in the promoter region in the *seb1-1* strain, and we plotted it as a metaprofile (Fig. 3B, left panel). As a comparison, we present a group of genes that showed a drop in the pausing index in the promoter region (Fig. 3B, right panel). Nevertheless, in both gene classes, there is an additional peak around the PAS region, suggesting that Pol II accumulates at the 3′-end for both gene groups in *seb1-1* strains.

To further explore the correlation between Seb1 and pausing, we decided to stratify genes into two categories depending on whether the gene had Seb1 cross-linking in the promoter as found in Seb1 PAR-CLIP data (Wittmann et al. 2017). Next, we plotted the mean Pol II PRO-seq or NET-seq signal and noted that on average genes with Seb1 bound to RNA at the 5′-end of transcription show changes in pausing close to TSS (Fig. 3C, right panel; Supplemental Fig. 2A; Booth et al. 2016; Wittmann et al. 2017; Parsa et al. 2018). As a reference for PRO-seq, we present metaprofiles for all genes or genes split into four quantiles depending on the pausing index (Fig. 3C, left and middle panels). To exclude the possibility that correlation between elevated pausing in Seb1-promoter bound gene set is purely due to the differences in expression levels, we undertook two approaches. First, we normalized PRO-seq signal of each gene by its expression level (corresponding to the nascent RNA levels measured by the transient transcriptome sequencing [TT-seq], Supplemental Fig. 2B). Moreover, we selected a subset of genes matching expression levels of Seb1-bound genes (exemplary plot Supplemental Fig. 2C, left panel) and calculated the mean for control set. Despite the differences being less striking, Seb1-bound genes show statistically significant broader pausing pattern (Supplemental Fig. 2C, right panel). In summary, we propose that Seb1 can promote pausing at TSS, or alternatively, it might facilitate pause release of Pol II at genes that have a high level of paused Pol II. Taken together, we suggest that Seb1 is an elongation factor that can contribute to control of Pol II pausing in a context-dependent manner.

## DISCUSSION

The 3′-end processing and termination of Pol II generates functional RNA molecules and allows polymerase recycling. CID/CID–RRM factors are central to the coordination of the last transcriptional steps. Here, we focused on Seb1 as a model for the CID–RRM factors and tested its functions in the defined in vitro systems and observed that Seb1 can promote Pol II transcription. Moreover, we provide evidence that Seb1 prevents and helps to resolve TFIIS-sensitive pauses in vitro. We also uncovered that the Pol II CTD is not necessary for Seb1 stimulation, which points toward this repetitive region functioning as a recruitment platform for CID–RRM factors that can be less important in the in vitro settings where Seb1 amounts are saturating. However, these findings do not diminish the essential role of the Pol II CTD in vivo, where it remains critical for proper transcriptional regulation (Wittmann et al. 2017). Seb1 functions to prevent long-lived backtracked pauses possibly via its RRM domain found in contact with the Pol II core close to the RNA channel and/or RNA binding of the nascent RNA (Wittmann et al. 2017; Kecman et al. 2018). This may happen via binding the transcript emerging from Pol II RNA-exit channel and physical blocking of backward movement of the elongation complex, that is, backtracking. An allosteric effect on elongation complex can also take place, in the manner of antitermination factor p7 action on bacterial RNA polymerase, that biases elongation complex away from the backward movement (Yuzenkova et al. 2008). Importantly, such action of Seb1 may also stabilize some sequence-dependent pauses that do not rely on backtracking (Landick 2009). While Seb1 potentially can outcompete Spt5 for Pol II binding, we can envision the possibility of the coexistence of these factors during the elongation/initiation phase (Kecman et al. 2018). In fact, the modular structure of Spt5 would be compatible with rearrangements of interactions within Pol II core without the full dissociation of Spt5 from Pol II (Yanagisawa et al. 2024; Zeng et al. 2024; Kuś et al. 2025).

To complement our in vitro findings, we decided to revisit publicly available data to provide correlations between Seb1 RNA binding sites and Pol II pausing in vivo (Booth et al. 2016; Wittmann et al. 2017; Parsa et al. 2018). Although Seb1 was proposed to induce long-lived pauses in vivo, we observed that for a subset of genes, this protein has the opposite effects (Parsa et al. 2018). Therefore, Seb1 might exhibit context-dependent control of Pol II pausing. In addition, the accumulation of pauses close to the PAS in the *seb1-1* mutant is compatible with the idea that Seb1 might modulate CPF cleavage, and when this process is compromised, Pol II accumulates around this region. Furthermore, we noted that genes with Seb1 cross-linking in their promoter regions show different pausing propensity compared to genes lacking Seb1 PAR-CLIP signals. This correlation suggests two possible interpretations: either Seb1 directly promotes pausing potentially via binding to RNA, or alternatively, Seb1 preferentially targets already-paused genes to regulate their transition into elongation.

It may seem counterintuitive that Seb1 or Scaf4/8 might act as termination and elongation factors. Nevertheless, the dualism of action for transcriptional factors has been observed before including Spt5, which can promote pausing at TSS or act as a positive elongation factor (Wada et al. 1998; Bourgeois et al. 2002; Hu et al. 2021). In bacteria, NusG and NusA display opposing effects on termination depending on the external cues (Mooney et al. 2009; Wells et al. 2016). This plasticity may extend to Seb1, where its functionality might be modulated by the binding partners (RNA or CTD) and post-translational modifications, particularly phosphorylation of its multiple residues could serve as a molecular switch between pro- and antitermination states (Swaffer et al. 2018). Alternatively, the composition of the complexes associated with Pol II might dictate which role Seb1 takes in each transcriptional step. Therefore, integration of multiple signals might be required for Pol II to undergo 3′-end processing and termination. Seb1 could be a nexus for such regulation as it can form multivalent interactions with Pol II (CTD and core), nascent RNA but also with CPF complex. Although our data suggest that Seb1 can act as a positive elongation factor, further research is required to evaluate the impact of Seb1 on the transcriptional elongation. This could be achieved with rapid Seb1 depletion and measurements of nascent transcription like TT-seq.

In summary, we propose that Seb1 can control Pol II pausing and act as an elongation factor beyond its involvement in 3′-end processing/termination. These functionalities appear to be separated in higher eukaryotes, where gene duplication created paralogs (Scaf4 and Scaf8) that possess overlapping but also unique functions. Differences in genome organization and the length of the genes could have been a driving force for the separation of Seb1 homologs’ functions. Scaf4 seems to resemble more Seb1 3′-end functionalities, and both Scaf4/8 act as positive elongation factors (antiterminators). Overall, Seb1 might function as a context-dependent regulator of Pol II progression ascertaining that 3′-end processing and transcription termination would generate functional RNA molecules.

## MATERIALS AND METHODS

### Protein purification

Pol II was purified in a similar manner as previously described (Kecman et al. 2018). Briefly, *S. pombe* yeast strain with endogenously tagged Rpb9-3xFLAG (and TEV cleavage site before the CTD of Rpb1) was grown in YES media (genotype: h+, *leu1-32*, *ura4*-Δ18, *ade6*-M216; *his3*Δ::1; *rpb9*-3xFLAG::*kanMX*; *rpb1*–TEV–CTD—a TEV cleavage site was inserted upstream of the CTD), harvested, and stored at −80°C until the day of purification. Cells were lysed in a freezer mill (SPEX SamplePrep) in liquid nitrogen and mixed with lysis buffer [50 mM Tris-HCl pH 7.5, 150 mM NaCl, 10% glycerol, 0.5% Triton X-100, 0.5 mM DTT, 0.5 mM MgCl_2_, 0.5 mM Mg(OAc)_2_] supplemented with proteinase inhibitor cocktail (cOmplete, EDTA-free Protease Inhibitor Cocktail, Roche). Lysate was cleared using centrifugation at 40,000*g* for 20 min and incubated with α-FLAG beads (M2 agarose gel, Sigma-Aldrich) for 1.5 h in the cold room. Beads were washed three times with W1 buffer [50 mM Tris-HCl pH 7.5, 1 M NaCl, 1 M urea, 10% glycerol, 0.5% Triton X-100, 0.5 mM DTT, 0.5 mM MgCl_2_, 0.5 mM Mg(OAc)_2_] and three times with W2 (as W1 but 150 mM NaCl, no urea, 0.05% Triton X-100). Next, Pol II was eluted with 5 mL of 2.5 mg/mL 3xFLAG-peptide (Sigma-Aldrich) and mixed with 20 mL of Q_A buffer [50 mM TRIS pH 7.7, 5 mM NaCl, 10% glycerol, 0.5 mM MgCl_2_, 0.5 mM Mg(OAc)_2_, 1 mM β-mercaptoethanol]. The protein eluate was then applied to an ion exchange chromatography column (2 × 1 mL or 1 × 5 mL HiTrap Q HP, GE Healthcare) equilibrated with Q_A buffer. The column was washed with several column volumes of 8% Q_B buffer (as Q_A but 2 M NaCl). The protein was eluted with a gradient of Q_B buffer (up to 40%). The buffer was exchanged to storage buffer [20 mM HEPES pH 7.5, 150 mM NaCl, 0.5 mM MgCl_2_, 0.5 mM Mg(OAc)_2_, 1 mM β-mercaptoethanol], and proteins were snap-frozen and kept at −80°C until use. To remove the CTD, Pol II with the TEV cleavage site was incubated with AcTEV (Invitrogen) at room temperature. Pol II with or without the CTD was subjected to gel filtration on Superose 6 3.2/300 (GE Healthcare), aliquoted, and stored at −80°C for further experiments.

Seb1 (full-length, with C-terminal His-tag, in pET41a plasmid) was expressed in Rosetta *E. coli* strain at 37°C using IPTG induction for 4 h. Cells were harvested by centrifugation at 4°C and frozen. Cell pellets were resuspended in NiTA buffer (50 mM Tris-HCl pH 7.7, 600 mM NaCl, 5 mM imidazole, 1 mM β-mercaptoethanol) with proteinase inhibitor mix. Cells were lysed using French Press, and phenylmethylsulfonyl fluoride (PMSF) was added to 1 mM final concentration. Lysates were spun at 20,000*g*, 4°C for 30 min, filtered, and incubated with Ni-NTA Agarose (QIAGEN, 1 mL slurry per 2 L of cell culture) for 1 h. Beads were washed with several column volumes of NiTA buffer, and protein was eluted with 500 mM imidazole. Next, the protein was centrifuged at 10,000*g* for 10 min and subjected to gel filtration on Superdex 200 10/300 (GE Healthcare) equilibrated with GF buffer [20 mM HEPES pH 7.6, 150 mM NaCl, 1 mM DTT, 0.5 mM MgCl_2_, and 0.5 mM Mg(OAc)_2_]. Protein was snap-frozen and kept at −80°C till use.

Truncated variant of TFIIS (115–293 amino acids of *S. pombe tfs1* gene, codon optimized for *E. coli*, as N-terminal His-tag, with TEV cleavage site) was purified in a similar manner as Seb1 except nickel affinity was performed on HisTrap HP (GE Healthcare), followed by ion exchange (HiTrap SP HP, GE Healthcare) and with final gel filtration on HiLoad 16/60 Superdex, 75 PG. The proteins were snap-frozen and kept at −80°C.

### In vitro transcription

Tailed DNA templates were prepared using PCR, cleaved with BsaI enzyme (New England Biolabs), dephosphorylated with Antarctic phosphatase (New England Biolabs), ligated with a single-stranded phosphorylated oligo (to create a 3′-end overhang on template strand), and purified using PCR Purification Kit (QIAGEN). Sequences are listed in Supplemental Table 1. Typically, a 20 µL starting reaction containing tailed DNA template (125–170 ng), 1 µL UpG (7.5 mM dinucleotide primer, Jena Bioscience), Pol II (250–400 ng) and 5 µL rNTPs (50 µM rATP, 50 µM rGTP, 5 µM rUTP with added 1 µL hot α-^32^P-rUTP 0.37 MBq/µL [Hartmann Analytic] without rCTP), RNasin (0.5 µL, Promega) was incubated in TB1 buffer [TB1: 37.5 mM HEPES pH 7.5, 150 mM KCl, 0.375 mM DTT, 0.75 mM MnCl_2_, 0.75 mM MgCl_2_, 0.75 mM Mg(OAc)_2_, 9% glycerol]. This allowed transcription of C-less segment, and reactions were carried out for 25–30 min at room temperature. Proteins (1/5 or 1/6 of reaction volume, final concentration indicated in the figure) were incubated with the complex (10–15 min), and rCTP (10–12.5 µM) was used to resume transcription. Samples were collected at indicated time points and stopped with the addition of 0.2 mg/mL proteinase K, 12 mM EDTA, and 0.2% SDS. RNA products were separated on 6% or 8% PAGE-urea gel and visualized on a FLA-7000 phosphoimager (Fujifilm) or autoradiography film (Amersham Hyperfilm MP).

Artificial elongation complexes were assembled as described by us earlier (Nielsen and Zenkin 2013; Heo et al. 2021). Briefly, reactions were assembled in a stepwise manner in TB buffer (20 mM Tris, 40 mM KCl, 200 µM EDTA, pH adjusted to 7.9 with HCl). First, for final standard 10 µL reaction, ∼2.5 pmol of 5′ radioactively labeled RNA was annealed with 1 pmol of template DNA at 45°C and slowly cooled to room temperature. Next, 0.3–0.4 pmol of Pol II was added to the mix and kept for 10 min at 30°C, followed by the same incubation with 10 pmol of nontemplate DNA (Supplemental Table 1, T-DNA, NT-DNA, RNA13 labeled with ^32^P on 5′). Complex was immobilized on equilibrated streptavidin beads (Streptavidin Sepharose HP, Cytiva, 6 µL per reaction) as previously reported (Nielsen et al. 2013). Beads were washed once with TB, once with W500 (20 mM TRIS, 500 mM NaCl pH adjusted to 7.9 with HCl), and again three times with TB. After that, the complex was incubated with buffer or protein at the concentration specified in the figure legend for at least 5 min at 30°C. Transcription was started with the addition of rNTPs/Mg^2+^ at the concentration indicated in the figure legend, and samples were collected at the times specified (reaction stopped with an equal volume of formamide containing 20 mM EDTA, 7 M urea, 100 μg/mL heparin, 0.02% bromophenol blue, 0.03% xylene cyanol in formamide). In experiments shown in Figure 2C,D and Supplemental Figure 1C, proteins were supplemented with 3 mM EDTA to inhibit TFIIS activity until transcription is initiated. Products were resolved on 10% PAGE-urea gel and visualized on a FLA-7000 phosphoimager (Fujifilm). Reactions were performed at least twice (with the same or different templates).

### Bioinformatics

For the analysis, only coding genes were considered, and it was required that the gene did not have another transcription unit annotated on the same strand within 250 bp before TSS or after PAS (*n* = 3190). Publicly available NET-seq data (accession: GSE114540) in bigwig format was used to calculate the pausing index. Only genes longer than 900 bp and having signal >10 in the gene body were used. The pausing index was defined as the ratio of the signal in 300 bp downstream from TSS, divided by read density in the gene body (region TSS+300 bp to PAS-300 bp). To select genes with increased pausing index in the *seb1-1* strain, we required that the trend is consistent between the replicates with a difference in pausing index of at least 0.5. Most likely, our analysis underestimates the number of genes with increased/decreased pausing as we limited our analysis to a subset of genes. Metaprofiles were prepared for each replicate using deepTools (Ramírez et al. 2016), averaged, and plotted in R package (Ihaka and Gentleman 1996).

To correlate Seb1 RNA binding and pausing, we used PAR-CLIP data (accession: GSE93344) (Wittmann et al. 2017) and PRO-seq data (accession: GSM1974985) (Booth et al. 2016). The pausing index was defined as above except genes with any signal in the gene body and longer than 600 bp were considered (*n* = 3015). The gene body was defined as a region between TSS+300 bp and PAS. Next, genes were assigned according to the presence of the Seb1 cross-linking in the promoter. Metaprofiles (average of all genes, quantiles, and genes with/without Seb1 cross-links in the promoter region) were plotted in the R environment.

## SUPPLEMENTAL MATERIAL

Supplemental material is available for this article.

## Figures and Tables

**Krzysztof Kuś:**
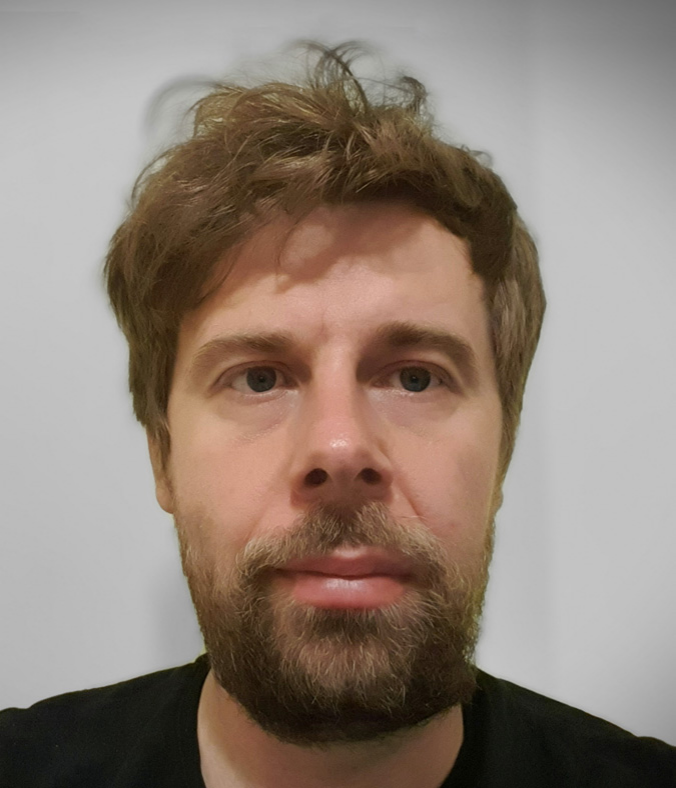


## MEET THE FIRST AUTHOR

***Meet the First Author(s)* is an editorial feature within *RNA*, in which the first author(s) of research-based papers in each issue have the opportunity to introduce themselves and their work to readers of *RNA* and the RNA research community. Krzysztof Kuś is the first author of this paper, “Conserved protein Seb1 that interacts with RNA polymerase II and RNA is an antipausing transcription elongation factor.” Krzysztof is a postdoctoral researcher in Lidia Vasilieva's laboratory in the Department of Biochemistry at the University of Oxford. Vasilieva's lab focuses on various aspects of transcriptional regulation, with a current emphasis on 3′ end processing and termination.**

**What are the major results described in your paper, and how do they impact this branch of the field?**

RNA polymerase II (Pol II) is responsible for the transcription of all protein-coding genes and many noncoding RNAs. Transcriptional regulation is a highly coordinated process involving numerous protein factors and post-translational modifications. This study focuses on Seb1 (in fission yeast, or Scaf4/8 in humans), a member of the CID–RRM family of Pol II regulatory proteins. These factors contain a CTD-interacting domain (CID) that binds to the C-terminal domain (CTD) of Pol II, and an RNA recognition motif (RRM) that mediates RNA binding. The CTD is composed of repeating heptapeptides whose phosphorylation pattern changes during transcription, serving as a dynamic platform for factor recruitment. CID–RRM proteins recognize these phosphorylation states, allowing them to engage with Pol II at specific transcriptional stages. Using biochemical and bioinformatic approaches, our study reveals that Seb1 regulatory functions extend far beyond its previously recognized roles. We demonstrate that Seb1 can directly stimulate Pol II activity and modulate polymerase pausing. Strikingly, our analyses suggest that in vivo, Seb1 can either enhance or suppress pausing, depending on the context. These findings highlight the dual nature of transcriptional regulators and substantially broaden our understanding of Seb1, transforming it from a factor primarily associated with 3′ end processing and termination into a multifaceted regulator potentially acting throughout the transcriptional cycle.

**What led you to study RNA or this aspect of RNA science?**

We find transcriptional regulation fascinating, particularly the intricate ways proteins exert their activity to control gene expression. What drew us to this work was the opportunity to use a minimalistic in vitro system to test how these proteins actually function. By stripping away the cellular complexity, we could directly observe and measure Seb1 activities in a controlled environment, allowing us to uncover mechanisms that might be obscured in more complex experimental settings.

**If you were able to give one piece of advice to your younger self, what would that be?**

Do not be afraid of failure. Science does not work in a linear way, and setbacks are an inherent part of the process. What matters most is persistence—the willingness to learn from unsuccessful experiments, adapt your approach, and keep moving forward.

**Are there specific individuals or groups who have influenced your philosophy or approach to science?**

No scientist works in isolation—“We all stand on the shoulders of those who came before us.” I have been directly inspired by both my former and current supervisors, who have shaped how I approach scientific questions and experimental design. In a broader context, I tend to think in Darwinian terms—that “nothing in biology makes sense except in the light of evolution.” This evolutionary perspective provides a framework for understanding why molecular mechanisms exist and how they have been refined over time, which continually influences how I interpret data and formulate hypotheses.

**What are your subsequent near- or long-term career plans?**

My aim is to transition toward independence and develop my own research program in the near future. I hope to build on the foundation I have established during my postdoctoral training to explore questions in transcriptional regulation. Understanding how regulatory factors operate in different contexts and coordinate with one another remains a rich area for discovery, and I am eager to pursue these questions using multidisciplinary approaches.
